# Solvent polarity mediates phytochemical yield and antioxidant capacity of *Isatis tinctoria*

**DOI:** 10.7717/peerj.7857

**Published:** 2019-10-09

**Authors:** Abdul Wakeel, Sohail Ahmad Jan, Ikram Ullah, Zabta Khan Shinwari, Ming Xu

**Affiliations:** 1 Key Laboratory of Geospatial Technology for Middle and Lower Yellow River Regions, School of Environment and Planning, Henan University, Kaifeng, Henan, China; 2 Molecular Systematics and Applied Ethnobotany Lab (MoSAEL), Department of Biotechnology, Quaid-i-Azam University, Islamabad, Pakistan; 3 Department of Biotechnology, Hazara University, Dodhial, Mansehra, Khyber Pakhtunkhwa, Pakistan; 4 Department of Ecology, Evolution and Natural Resources, Rutgers University, New Jersey—Camden, United States of America

**Keywords:** Solvent polarity, Antioxidant activity, *Isatis tinctoria*, Phenolic content, Flavonoid content

## Abstract

Secondary metabolites have been extensively used in the treatment of various health problems. The role of solvent polarity on the phytochemical isolation and antioxidant capacity of *Isatis tinctoria* (woad) is elusive. In the present study, 14 solvents with different polarity were used in the extraction and total phenolic and flavonoid content (TPC and TFC) investigation. Ferricyanide, phosphomolybdenum, and 2,2-diphenyl-1-picrylhydrazyl (DPPH) methods were used to calculate and compare the antioxidant/free radical scavenging capacity. Our results showed that solvent polarity greatly affects TPC and TFC yield, which is mainly increasing with increasing solvent polarity index and suddenly decreasing at very high polarity. The comparative results showed that TPC is directly correlated with reducing power, antioxidant, and free radical scavenging capacity. Taken together, we conclude that different woad plant parts contain different level of secondary metabolites with a specific polarity that requires a particular solvent with an appropriate polarity index for the extraction. The identification of these biologically active crude extracts and fractions are very important for the basic biological sciences, pharmaceutical applications, and future research for HPLC based active compounds isolation.

## Introduction

Plants are excellent source of secondary metabolites such as phenolics, flavonoids, alkaloids, lignans, and terpenoids. Secondary metabolites have been extensively used since ancient times and are still very popular in the treatment of various diseases and disorders (Karakaya et al., 2019). In plants, reactive oxygens species (ROS) balance is disturbed by the exogenous/endogenous stimuli that might cause various ultrastructural damages, protein and chromosome alterations, and DNA single and double-strand breakages (Hu, Cools & De Veylder, 2016; Jia, Liu & Gao, 2016; Wakeman et al., 2017). Plants synthesize secondary metabolites for scavenging excessive amounts of ROS and free-radicals to cope with the possible damage. Primarily, plants produce these secondary metabolites (phenols, flavonoids, and tannins) for their own defense, which can be used for the treatment of other living organism facing ROS-mediated chromosomal, ultrastructural, DNA damages, and protein denaturations and deactivation at both translational and post-translational levels (Rai & Mehrotra, 2008; Hu, Cools & De Veylder, 2016; Mikulášová, 2019). Scientists are gaining more interest in the medicinal plant-derived diverse group of compounds with a broad range of applications.

Medicinal plants have a wide range of phytochemicals and secondary metabolites that are used in a range of biomedical and industrial applications (Ullah et al., 2017). For the green pharmaceuticals, scientists prefer plants that have a history of medicinal uses. These plants are explored for crude extracts, extracts fractions, and specific compound isolation. These compounds can be used as a precursor for the synthesis of allopathic drugs (Makkar, Siddhuraju & Becker, 2007; Ncube, Afolayan & Okoh, 2008). Literature reports that these phytochemicals have synergistically increased the efficacy of synthetic drugs and can be used with the allopathic drugs for the treatment of health problems (Güner et al., 2019). HPLC based analysis of the medicinal plants has reported and commercialized life saving therapeutics such as tryptanthrine and artemisinin (Mohn, Plitzko & Hamburger, 2009; Güner et al., 2019; Zafar et al., 2019). The quality, quantity, and biological activities of these phytochemicals are directly dependent on the plant developmental stage, plant parts, and the solvents used for the extraction and isolation (Senguttuvan, Paulsamy & Karthika, 2014; Ullah et al., 2017; Chekroun-Bechlaghem et al., 2019). In the current study, branches, flowers, leaves, and roots of *Isatis tinctoria* (woad) were selected on the bases of their use in the folk/conventional medicine.

Woad belongs to the family *Brassicaceae* commonly found in the northwest region of Pakistan, Iran, Mongolia, Uzbekistan, Tajikistan, Kazakhstan, Japan, Korea, Russia, South-west Asia, Europe, and United States of America (Hamburger, 2002; Ullah et al., 2017). Different parts of the woad plant have been used in folk medicine, as a powder or crude water extract. Lipophilic extracts of woad have shown anti-inflammatory responses. Lipophilic woad extract *in vivo* studies strongly supports anti-inflammatory response such as in skin erythema (Forner et al., 2019; Marcelo, Gontier & Dauwe, 2019). The selection of solvents for extraction plays an important role in the quantity and quality of extracts.

Solvent type and polarity can affect the extract quality, quantity, extraction velocity, inhibitory compounds, toxicity, other biological activity, and biosafety (Eloff, 1998; Zhang et al., 2019). The total secondary metabolites and their antioxidant capacity greatly depend on the solvent and plant part used for extraction (Rafińska et al., 2019). In the present study, seven different organic solvents and their 1:1 (v/v) ratio combinations were employed based on the polarity index and **solvent miscibility** according to HPLC Solvent Guide, Solvent Miscibility and Viscosity Chart adapted from Paul Sadek, 2002, which was previously adapted by various researchers (Menkiti, Agu & Udeigwe, 2015; Ullah et al., 2017; Chinchu & Kumar, 2018). After the extraction, extraction efficiency, total phenolic content (TPC), total flavonoid content (TFC), ferricyanide, phosphomolybdenum, and 2,2-diphenyl-1-picrylhydrazyl (DPPH) method-based antioxidant/free radical scavenging activity were performed. Furthermore, Pearson’s correlation was performed using Minitab 16 software for the possible correlations.

## Material and Methods

### Sample collection

*Isatis tinctoria* (woad) plants were collected from the mountains of district Lower Dir (34°50′43.19″N, 71°54′16.43″E), Khyber Pakhtunkhwa (KP), Pakistan in March 2014. This district was selected because of the natural habitat for the woad plants. Plants were immediately transferred to Molecular Systematics and Applied Ethnobotany Lab (MOSAEL) at Quaid-I-Azam University (QAU), Islamabad, Pakistan. By the help of taxonomist at QAU, the plants were confirmed as *Isatis tinctoria* (Ullah et al., 2017). The plants were washed with tap water to remove the dust. Four parts woad plant (branches, flowers, leaves, and roots) were separately shade-dried (indoor not exposed to sunlight) at room temperature, ground into a fine powder by using FGHGF 2,500 g grinder. The powder sifting was done via strainer (2 mm pore size) for uniform particle size and stored at 4 °C.

### Solvent selection and combination

Solvents (n-hexane, chloroform, ethyl acetate, acetone, ethanol, methanol, and water) were selected and a combination (1:1, v/v, n-hexane-ethyl acetate, n-hexane-ethanol, methanol-chloroform, methanol-ethyl acetate, methanol-acetone, acetone-water, and methanol-water) of these solvents were employed for the extraction. These solvents were selected and combined on the bases of solvent polarity index and **miscibility** according to HPLC Solvent Guide, Solvent Miscibility and Viscosity Chart adapted from Paul Sadek in 2002, which was previously adapted by various researchers (Menkiti, Agu & Udeigwe, 2015; Ullah et al., 2017; Chinchu & Kumar, 2018).

### Extraction

For the extraction efficiency, 20 g powder of branches, flowers, leaves, and roots were separately soaked in 1 L canonical flasks containing 500 mL solvent (14 different flasks with different solvent for each plant part). The flasks, containing soaked plant powder, were sealed with a cotton plug and aluminum foil and placed on a shaker (BT1010 Benchmark Scientific Orbi-Shaker XL, NJ-USA) at room temperature for 24 h. After 24 h of incubation in the shaker, the flasks were transferred to a sonicator (SONICA Ultrasonic Sonication, Meizhou, Guangdong, China) for 5 min. The sonicated suspension was strained through a sterilized cheesecloth followed by filtration through Whatman filter paper grade 1. The filtrate was first evaporated using rotary evaporator (R-300 Rotary Evaporator; Buchi, Flawil, Switzerland), followed by vacuum drying at 0.06 atm pressure (Rocker 300C Vacuum Pump; Rocker, Kaohsiung, Taiwan) at room temperature.

The extraction efficiency of the solvent was calculated using the formula;

\begin{eqnarray*}\text{%} \mathrm{efficiency}= \left[ \frac{W2-W1}{W3} \right] \times 100 \end{eqnarray*}

where,

W1 = Weight of empty bottle

W2 = weight of bottle + Extract

W3 = Weight of powder used for extraction

Four milligrams of each extract was dissolved in 1 mL DMSO (Dimethyl sulfoxide) for further analysis.

### Total phenolic content (TPC) assay

To investigate the role of solvent polarity on the TPC, 20 µL of each sample (14 different extracts from branches, flowers, leaves, and roots each, 400 µg/mL DMSO), 20 µL of gallic acid (1 mg/mL, as positive control), and 20 µL DMSO and methanol (as a negative control) were added to 96-wells microplates, followed by the addition and mixing of 90 µL of the Folin-Ciocalteu’s reagent (10 times diluted, 100 mmol/L) to each plate via multi-channel micropipette. The plates were incubated at room temperature for 5 min. Finally, 90 µL sodium carbonate (7% w/v) was added to each well and mixed properly followed by 90 min incubation at room temperature (Chandra et al., 2014). Readings were carried out at 630 nm wavelength via microplate reader (Biotek ELX 800; Biotek, Winooski, VT, USA). Results were calculated and expressed as gallic acid equivalent (GAE) µg/mg of extract (Al-Duais et al., 2009).

### Total flavonoids content (TFC) assay

To investigate the role of solvent polarity on the TFC, aluminum chloride colorimetric method (Chang et al., 2002) was modified for the microplate method. Briefly, 20 µL of each sample (400 µg/mL), 20 µL of quercetin (1 mg/mL, as positive control), and 20 µL DMSO and methanol (as negative) were added to 96 wells plate, followed by the addition and mixing of 10 µL of aluminum chloride (10 g/L) in distilled water, 10 µL of potassium acetate (98.15 g/L) and 160 µL of distilled water via multi-channel micropipette. The plates were incubated at 37 °C for 30 min. The readings were taken at 450 nm via microtiter plate reader (Biotek ELX800; Winooski, VT, USA). The results were expressed as quercetin equivalent (QE) µg/mg of extract.

### Ferric reducing antioxidant power (FRAP) assay

To investigate the impact of solvent polarity on the total reducing power of extracts, potassium ferricyanide trichloroacetic acid method was used (Benzie & Strain, 1996) with some modifications and adaptation for microplate method (Athamena et al., 2019). Ferric reducing antioxidant power (FRAP) will be written as total reducing power (TRP) beyond this point. Eppendorf tubes were labeled, 40 µL sample was added to each tube followed by 50 µL (0.2 mol/L) sodium phosphate dihydrate (Na_2_HPO_4_. 2H_2_O) buffer, 50 µL 1% potassium ferricyanide (K_3_Fe (CN)_6_), and 50 µL 10% trichloroacetic acid. The mixture was centrifuged at 3,000 rpm for 10 min. After centrifugation 166.66 µL of the supernatant of each sample were added to 96 well plates followed by 33.3 µL ferric chloride (FeCl_3_, 1%). The readings were taken at 630 nm via microtiter plate reader (Biotek ELX800; Biotek, Winooski, VT, USA). DMSO was used as negative control and ascorbic acid (1 mg/mL) as a positive control. Results were expressed as ascorbic acid equivalent (AAE) µg/mg of extract.

### Total antioxidant capacity (TAC) assay

To evaluate the possible role of solvent polarity in the TAC of the extracts, the phosphomolybdenum method (Benzie & Strain, 1996) was used with some modifications and adaptations to microplate method (Lahmass et al., 2018). Microplates were labeled accordingly, followed by the addition 20 µL of the samples, 20 µL ascorbic acid (as a positive control), 20 µL DMSO each in different wells. All the wells were loaded with 180 µL of the reagent (0.6 mol/L H_2_SO_4_, 28 mmol/L NaH_2_PO_4_, 4 mmol/L ammonium molybdate). The plates were covered and incubated at 95 °C for 60 min in a water bath. The sample was transferred to other plates after cooling. The readings were taken at 695 nm via microtiter plate reader (Biotek ELX800). Values were expressed as ascorbic acid equivalent (AAE) µg/mg of extracts (Lahmass et al., 2018).

### DPPH free radical scavenging assay

To analyze the possible free radical scavenging capacity of all extracts, which are extracted with different polarity solvents and from different plant parts, the scavenging of free radical 2,2-diphenyl-1-picrylhydrazyl (DPPH), was investigated using 96-well microplates read by a microplate reader (Beara et al., 2009; Zhu et al., 2018). For the estimation of IC_50_ values, four different concentrations (200 µg/mL, 100 µg/mL, 50 µg/mL, and 20 µg/mL) of each extract were prepared and 5 µL were loaded in wells of the microplates. All wells containing 5 µL sample, 5 µL DMSO, and 5 µL methanol as negative control were loaded with 195 µL of freshly prepared DPPH solutions (25 µg/mL) and mixed by pipetting thoroughly. Plates were incubated in the dark at room temperature for 30 min. Readings were taken at 515 nm via microtiter plate reader (BioTek Elx800). DPPH free radical scavenging percentage was identified for each concentration and log IC_50_ values were calculated for all extracts (Fezai, Mezni & Rzaigui, 2018; Zhu et al., 2018).

### Statistical and graphical analysis

The current investigation was performed in triplicate, with at least two similar repeats. The basic analysis and the graphs were prepared using MS Excel. The figures were prepared using MS PowerPoint. The different letters indicate a significant difference on the basis of one-way ANOVA with Tukey’s test. ANOVA and the Pearson’s correlation were performed using Minitab 16 software (Wakeel et al., 2018a; Wakeel et al., 2018b.).

**Figure 1:**
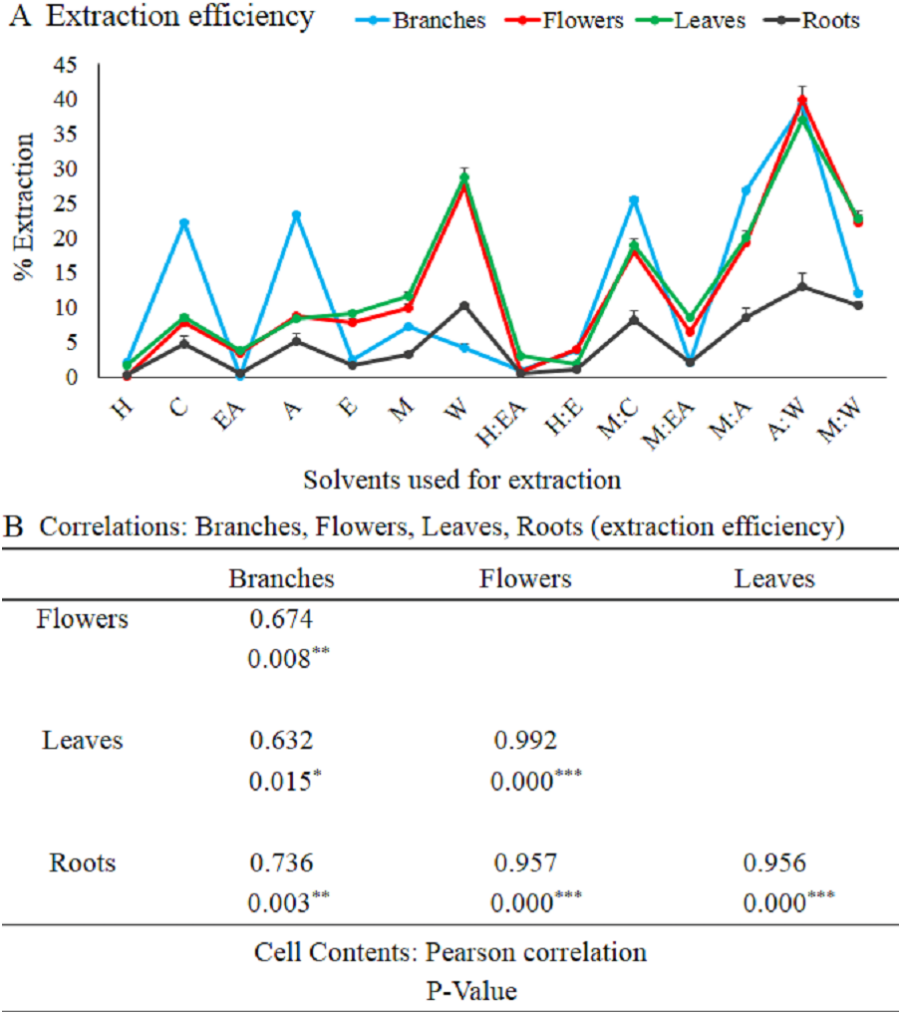
Polarity influences extraction efficiency in branches, flowers, leaves, and roots. The full form of the abbreviation used in the table: H (n-hexane), C (chloroform), EA (ethyl-acetate), A (acetone), E (ethanol), M (methanol), W (water), H:EA (n-hexane-ethyl-acetate), H:E (n-hexane-ethanol), M:C (methanol-chloroform), M:EA (methanol-ethyl-acetate), M:A (methanol-acetone), A:W (acetone-water), and M:W (methanol-water). Data shown are means ± STD (A). Correlations: branches, flowers, leaves, and roots (extraction efficiency). *p < 0.05, **p < .01, ***p < .001, Pearson’s correlation in Minitab 16 software (B).

## Results

### Extraction efficiency

The crude extract quantity, purity, and quality greatly depend on the plant part used and the solvent used for the extraction (Ullah et al., 2017; Jacotet-Navarro et al., 2018). To investigate the impact of solvent type and plant part on the bioactive extraction process, seven solvents (n-hexane, chloroform, ethyl acetate, acetone, ethanol, methanol, and water) were selected. Furthermore, to achieve more variety in polarity index, one ratio one (1:1, v/v) combinations of solvents (n-hexane-ethyl acetate, n-hexane-ethanol, methanol-chloroform, methanol-ethyl acetate, methanol-acetone, acetone-water, and methanol-water) were also prepared. Different solvents extracted different quantity of crude extract from branches, flowers, leaves, and roots. The overall extraction efficiency of branches was higher and lower in roots as compared to flowers and leaves. The maximum extraction efficiency for flowers, branches, leaves, and roots was 40, 39, 37 and 13% respectively (Fig. 1A). The extraction efficiency of acetone-water (1:1, v/v) was very high and ethyl acetate, n-hexane alone or in combinations was very low for all plant parts (branches, flowers, leaves, and roots) as compared to other solvents (Fig. 1A). Although the amount of crude extract was different in each part, there was a significantly positive correlation between branches, flowers, leaves, and roots based on the Pearson’s correlation (Fig. 1B).

**Figure 2:**
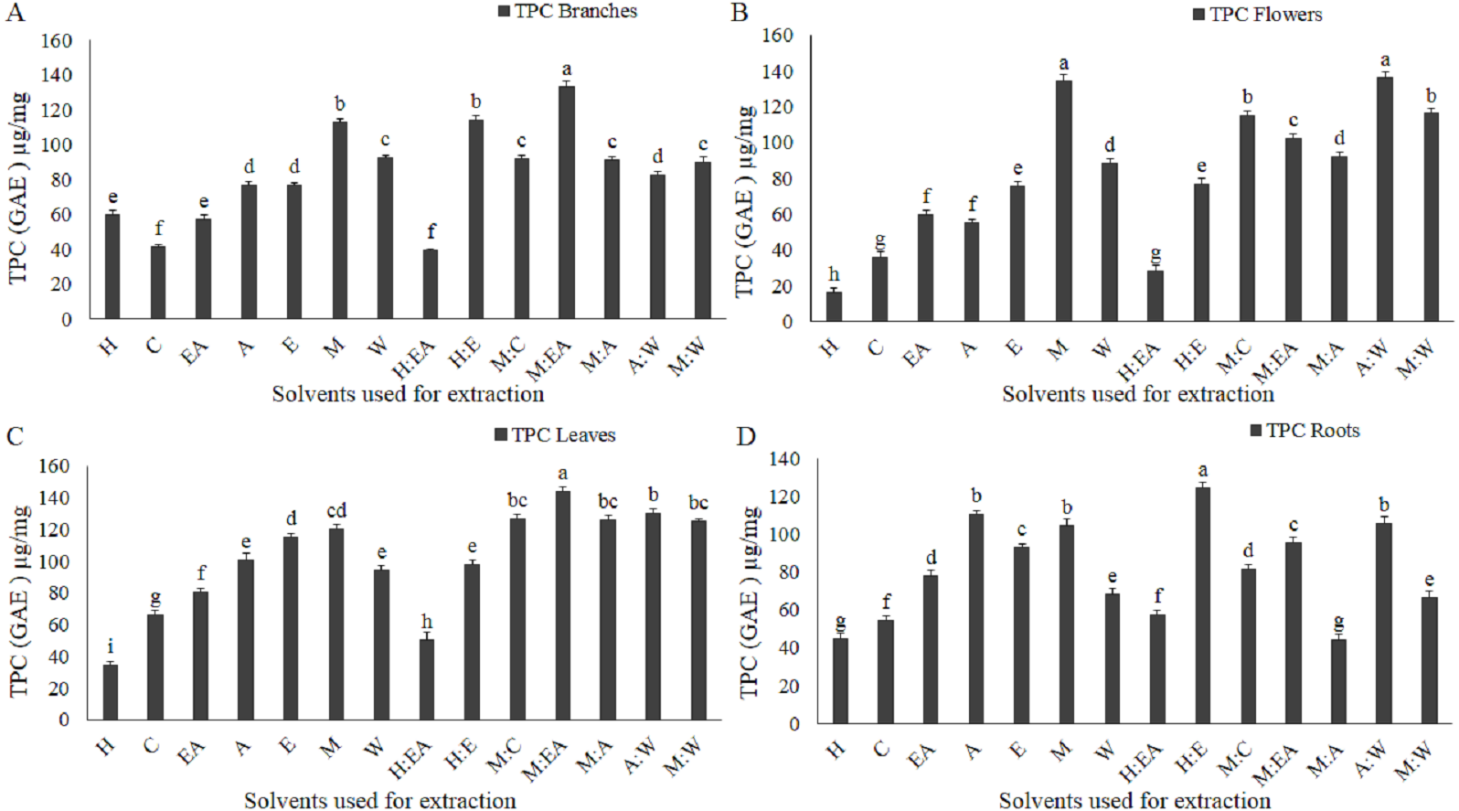
Total phenolic content (TPC) as µg/mg Gallic acid equivalent (GAE) is greatly modulated by the solvent type and polarity. µg/mg GAE TPC in branches (A), flowers (B), leaves (C), and roots (D). The full form of the abbreviation used in the table: H (n-hexane), C (chloroform), EA (ethyl-acetate), A (acetone), E (ethanol), M (methanol), W (water), H:EA (n-hexane-ethyl-acetate), H:E (n-hexane-ethanol), M:C (methanol-chloroform), M:EA (methanol-ethyl-acetate), M:A (methanol-acetone), A:W (acetone-water), and M:W (methanol-water). Data shown are means ± STD. Different letters indicate a significant difference between different extracts (*p* < .05 by one-way ANOVA with Tukey’s test using Minitab 16 software).

### Total phenolic contents (TPC)

Solvent type and polarity play an important role in TPC. Different plant parts have a different level of TPC (Lesjak et al., 2011). The extractions were carried out with 14 different solvents (Figs. 2A–2D). TPC level significantly increased with increasing solvent polarity with a few exceptions. Methanol-ethyl acetate in branches and leaves, methanol and acetone-water in flowers, n-hexane-ethanol in roots were the most efficient solvents (Figs. 2A–2D). Chloroform, n-hexane, and n-hexane-ethyl acetate showed the minimum level of TPC in all four parts (Figs. 2A–2D).

**Figure 3:**
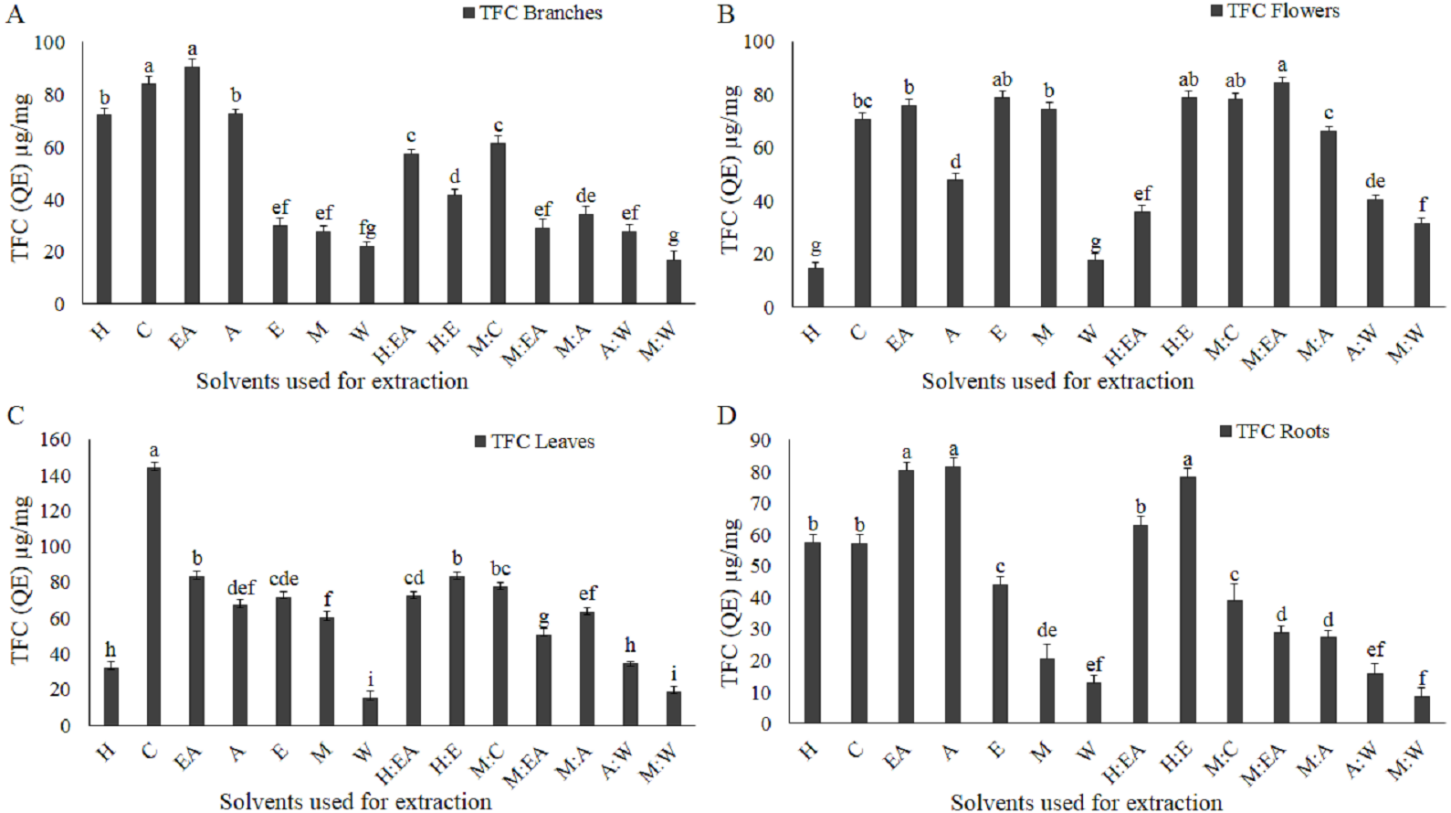
Total flavonoid content (TFC) as µg/mg quercetin equivalent (QE) is influenced by the solvent type and polarity. Textmug/mg QE TFC in branches (A), flowers (B), leaves (C), and roots (D). The full form of the abbreviation used in the table: H (n-hexane), C (chloroform), EA (ethyl-acetate), A (acetone), E (ethanol), M (methanol), W (water), H:EA (n-hexane-ethyl-acetate), H:E (n-hexane-ethanol), M:C (methanol-chloroform), M:EA (methanol-ethyl-acetate), M:A (methanol-acetone), A:W (acetone-water), and M:W (methanol-water). Data shown are means ± STD. Different letters indicate a significant difference between different extracts (*p* < .05 by one-way ANOVA with Tukey’s test using Minitab 16 software).

**Figure 4:**
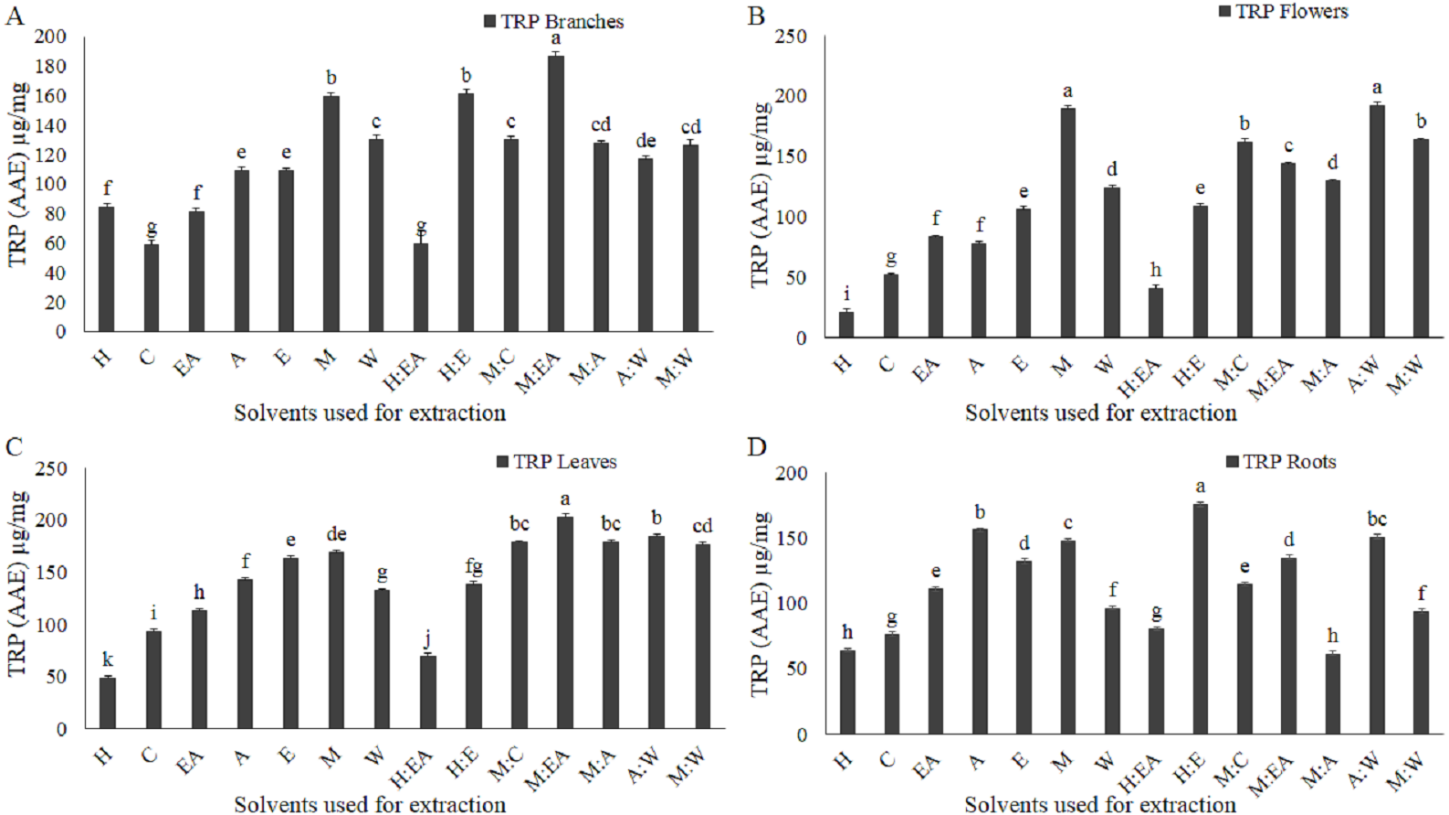
Total reducing power (TRP, potassium ferricyanide-ferric trichloroacetic acid method) as µg/mg ascorbic acid equivalent (AAE) was altered by the solvent type and polarity. µg/mg AAE TRP of the extracts, extracted from branches (A), flowers (B), leaves (C), and roots (D). The full form of the abbreviation used in the table: H (n-hexane), C (chloroform), EA (ethyl-acetate), A (acetone), E (ethanol), M (methanol), W (water), H:EA (n-hexane-ethyl-acetate), H:E (n-hexane-ethanol), M:C (methanol-chloroform), M:EA (methanol-ethyl-acetate), M:A (methanol-acetone), A:W (acetone-water), and M:W (methanol-water). Data shown are means ± STD. Different letters indicate a significant difference between different extracts (*p* < .05 by one-way ANOVA with Tukey’s test using Minitab 16 software).

**Figure 5:**
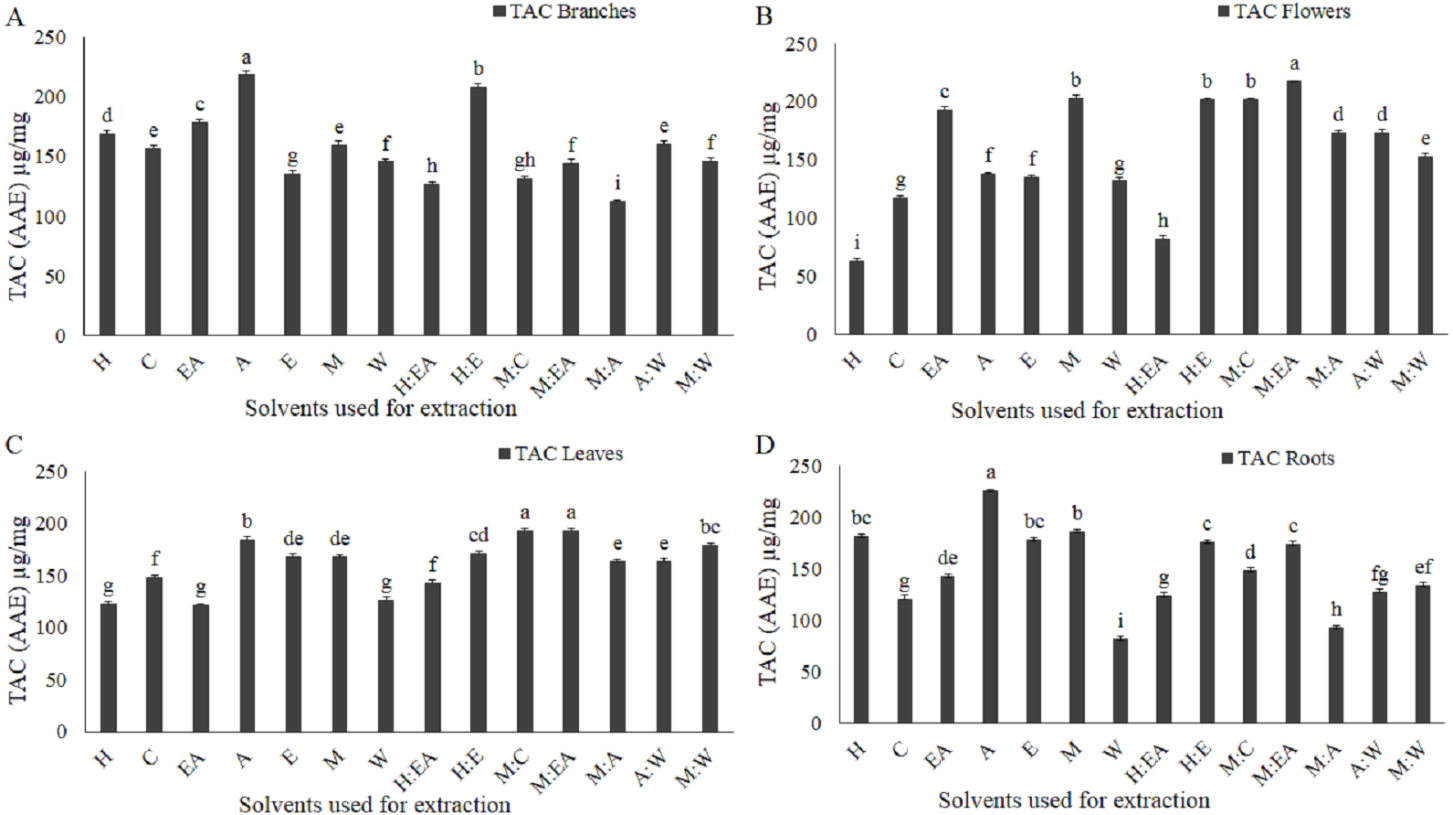
Total antioxidant activity (TAC, phosphomolybdenum method) as µg/mg ascorbic acid equivalent (AAE) was markedly influenced by the solvent type and polarity. µg/mg AAE TAC of the extracts, extracted from branches (A), flowers (B), leaves (C), and roots (D). The full form of the abbreviation used in the table: H (n-hexane), C (chloroform), EA (ethyl-acetate), A (acetone), E (ethanol), M (methanol), W (water), H:EA (n-hexane-ethyl-acetate), H:E (n-hexane-ethanol), M:C (methanol-chloroform), M:EA (methanol-ethyl-acetate), M:A (methanol-acetone), A:W (acetone-water), and M:W (methanol-water). Data shown are means ± STD. Different letters indicate a significant difference between different extracts (*p* < .05 by one-way ANOVA with Tukey’s test using Minitab 16 software).

### Total flavonoid contents (TFC)

Solvent type and polarity index, plants species and plant parts play an important role in the TFC level in the extracts (Do et al., 2014). In contrast, to the TPC level, the TFC level was quite low and it was not dependent on the solvent polarity (Figs. 3A–3D). Based on the TFC level, chloroform and ethyl acetate in branches (Fig. 3A); methanol-ethyl acetate, methanol-chloroform, and n-hexane-ethyl acetate in flowers (Fig. 3B); chloroform in leaves (Fig. 3C); and ethyl acetate, acetone, and n-hexane-ethanol in roots (Fig. 3D) were the most efficient solvents. The extracts, extracted with water and methanol-water were found to be the most inefficient solvent with minimum TFC level in all plant parts (Figs. 3A–3D). Except for chloroform extract in leaves, which is 143 µg/mg QE (Fig. 3C), the rest of the plant parts showed less than 90 µg/mg QE (Figs. 3A, 3B, and 3D).

### Ferric reducing antioxidant power

The solvent type and polarity, as well as plant parts, play an important role in the total reducing power (Do et al., 2014; Ullah et al., 2017). The TRP level increased with increasing solvent polarity index, except for water, which has the maximum polarity index among the selected solvents (Figs. 4A–4D). Methanol-ethyl acetate in branches and leaves; methanol and acetone-water in flowers; and n-hexane-ethanol in roots were the most efficient solvents (Figs. 4A–4D). Chloroform, n-hexane, n-hexane-ethyl acetate were the most inefficient solvents based on the total reducing power in all plant parts used in the current study (Figs. 4A–4D).

### Total antioxidant capacity (TAC)

The method of extraction, selection of plant parts, and use of appropriate solvent for the extraction greatly influence antioxidant capacity (Musa et al., 2011). In the current study, phosphomolybdate method was adapted to evaluate the impact of solvent polarity on the total antioxidant capacity in four different parts of woad. Phosphomolybdate method is adopted by a large group of researchers for the evaluation of antioxidant capacity and is considered one of the most authentic methods used for TAC (Chekroun-Bechlaghem et al., 2019). TAC of extracts varied significantly among different solvents and plant parts, which indicates that each solvent with specific polarity can isolate specific compounds that have a specific antioxidant capacity. Acetone in branches and roots (218 µg/mg and 226 µg/mg respectively, Figs. 5A and 5D), methanol-ethyl acetate in flowers and leaves (217 µg/mg and 193 µg/mg respectively, Figs. 5B–5C), and methanol-chloroform in leaves (193 µg/mg, Fig. 5C) showed the maximum TAC.

### DPPH free radical scavenging capacity

Extraction techniques, solvent type, and polarity, plant part selected for extraction mediate antioxidant and free radical scavenging capacity (Chekroun-Bechlaghem et al., 2019). DPPH free radical method has been extensively used for different natural and synthetic compounds antioxidant and free radicals scavenging potential (Zhu et al., 2018; Chekroun-Bechlaghem et al., 2019). First of all, the percent inhibition of DPPH free radicals was identified in four different concentrations (200, 100, 50, and 20 µg/mL) of each extract. Based on the percent inhibition log IC_50_ values were calculated for each solvent in the respective plant parts. The log IC_50_ value was significantly different in all solvents, which indicates that each solvent extracted some specific type of secondary metabolites that could carry different scavenging level of DPPH free radicals. Methanol in branches, ethyl acetate in flowers and leaves, and methanol-water in roots showed strong activity with minimum log IC_50_ values (0.2, 0.04, 0.09 and 0.04 µg/mL respectively, Table 1).

### Correlation

**Figure 6:**
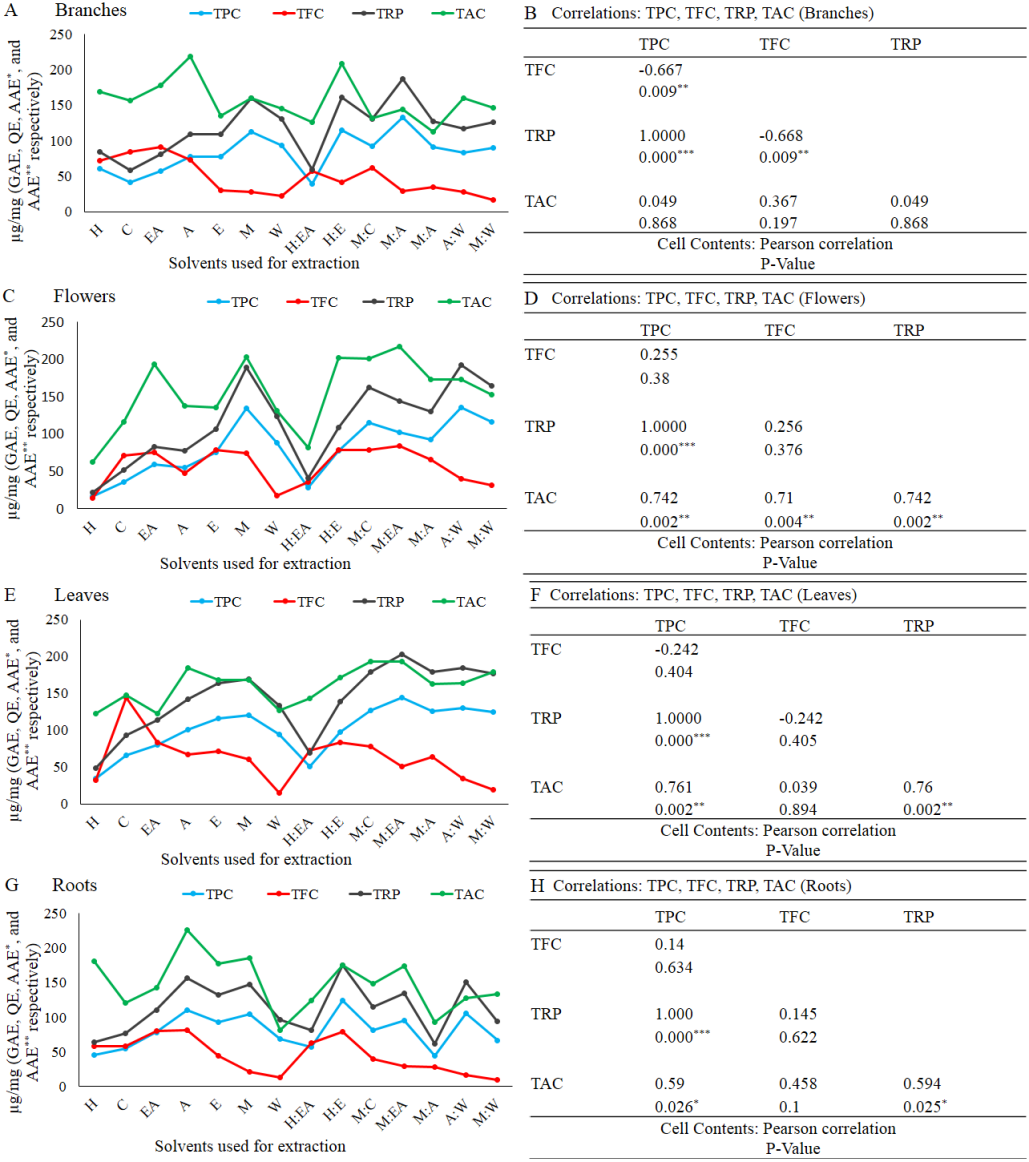
Total phenolic content is positively correlated with TRP and TAC in branches (A and B), flowers (B), leaves (C), and roots (D). The full form of the abbreviation used in the table: H (n-hexane), C (chloroform), EA (ethyl-acetate), A (acetone), E (ethanol), M (methanol), W (water), H:EA (n-hexane-ethyl-acetate), H:E (n-hexane-ethanol), M:C (methanol-chloroform), M:EA (methanol-ethyl-acetate), M:A (methanol-acetone), A:W (acetone-water), and M:W (methanol-water). Data shown are means ± STD. Correlations: TPC, TFC, TRP, and TAC in branches (B), flowers (D), leaves (F), and roots (H). *p < 0.05, **p < .01, ***p < .001, Pearson’s correlation in Minitab 16 software (B).

The Pearson’s correlation was performed for the relationship between TPC, TFC, TAC, and TRP in each plant part (branches, flowers, leaves, and roots) investigated in the current study. In branches, TPC showed significantly positive correlation with TRP, insignificant correlation with TAC, while significantly negative correlated with TFC. The correlation between TFC and TAC and TRP and TAC was insignificant, while the TFC and TRP was significantly negative correlated (Figs. 6A and 6B). In flowers, TPC showed significantly positive correlation with TRP and TAC, while insignificant correlation with TFC. The correlation of TFC with TAC was significantly positive, while with TRP it was insignificant. There was insignificant correlation between TAC and TRP (Figs. 6C and 6D). In leaves, the correlation of TPC with TRP and TAC was significantly positive, while with TFC, it was significantly negative. TFC showed significantly negative correlation with TRP, while no correlation with TAC. TRP and TAC showed significantly positive correlation (Figs. 6E and 6F). Finally, in roots, the TPC exhibited significantly positive correlation with TRP and TAC, while insignificant correlation with TFC. The correlation of TFC with TRP and TAC was insignificant. TRP showed significantly positive correlation with TAC (Figs. 6G and 6H). Taken together, it is concluded that TPC play an important role in the antioxidant capacity of woad plant extracts.

**Table 1:** The % inhibition and log IC_50_ values in different concentration for 14 different extracts of branches, flowers, leaves, and roots. The full form of the abbreviation used in the table: H (n-hexane); C (chloroform); EA (ethyl-acetate); A (acetone); E (ethanol); M (methanol); W (water); H:EA (n-hexane-ethyl-acetate); H:E (n-hexane-ethanol); M:C (methanol-chloroform); M:EA (methanol-ethyl-acetate); M:A (methanol-acetone); A:W (acetone-water); and M:W (methanol-water).

%Free radical scavenging and log IC50 in µg/ml of extracts concentration																					
	Plant Parts used	Branches					Flowers					Leaves					Roots				
	Conce (µg/ml)	200	100	50	20	IC50	200	100	50	20	IC50	200	100	50	20	IC50	200	100	50	20	IC50
Extracts with different solvents	H	81	75	72	71	1.08	86	78	72	70	1.392	79	70	73	75	1.60	92	74	72	71	2.54
	C	82	72	68	68	2.09	77	70	68	67	0.248	73	67	70	68	2.67	74	68	68	66	0.21
	EA	70	67	67	68	3.45	72	70	67	66	0.043	86	78	77	75	0.09	91	77	75	70	2.40
	A	65	66	63	66	5.26	62	61	61	59	6.381	80	74	71	66	1.61	80	76	75	63	1.90
	E	69	66	65	66	1.13	64	64	60	59	0.96	84	64	73	78	1.92	90	77	74	74	0.66
	M	72	67	65	58	0.20	72	70	68	65	0.081	81	76	74	72	0.11	88	84	82	75	0.14
	W	82	72	67	61	6.34	80	74	70	66	1.299	72	66	70	69	1.08	90	79	73	54	13.83
	H:EA	75	72	66	64	0.86	85	75	75	68	1.211	87	71	73	69	1.48	79	77	77	59	4.28
	H:E	78	71	69	67	2.63	88	80	75	68	2.573	79	73	69	65	1.98	89	66	65	55	16.41
	M:C	81	65	65	65	0.25	68	68	63	63	0.154	76	70	73	67	0.11	80	73	69	64	3.10
	M:A	76	72	69	67	1.66	79	74	70	65	1.87	80	80	76	70	0.22	67	67	63	62	0.27
	M:A	77	73	72	70	0.37	71	69	66	69	1.173	78	72	68	69	0.29	71	71	62	54	11.10
	A:W	77	71	64	64	0.72	67	69	65	67	2.13	84	76	71	66	2.67	99	90	72	65	8.80
	M:W	85	75	74	68	1.40	84	74	78	68	0.854	85	75	73	68	1.76	94	86	83	82	0.04

## Discussion

Medicinal plants have a wide range of phytochemicals that are directly dependent on the plant developmental stage, plant parts, and the solvents used for the extraction and isolation of these phytochemicals (Senguttuvan, Paulsamy & Karthika, 2014; Ullah et al., 2017; Chekroun-Bechlaghem et al., 2019). In the current study, it is reported that the crude extracts quality, purity, and quantity greatly depend on the plant part used and the solvent used for the extraction. The most efficient plant in terms of crude extract quantity was branches. The order of the plant parts based on the yielded extract quantity was branches>leaves>flowers>roots. In terms of crude extract quantity all plant part used showed a significantly positive correlations as shown in the figure (Figs. 1A and 1B). Consistent with previously reported data that these plant parts yielded a significantly different amount of the TPC, TFC, TAC, and TRP (Do et al., 2014). These plant parts have also shown a divers crude extract quantiy as previously reported in an antibacterial investigation of woad plant crude extracts (Ullah et al., 2017). The second most important component in the extraction process is solvent type and polarity (Do et al., 2014; Rafińska et al., 2019). Acetone-water (1:1, v/v) was the most efficient solvent in terms of crude extract quantity. Current results, showed that polarity index play active role in the extraction process. The quantities of crude extracts with different solvents were different in different plant parts. Ullah et al. (2017) reported that the extracts of these solvents have significantly different antibacterial and antifungal activity. The different antimicrobial activities of these solvents and plants parts might be because of the different types and quantity of biological compounds in these extracts. The role of solvent polarity in the quantity and quality of crude extracts, secondary metabolites, and biological activities has been previously reported (Do et al., 2014; Rafińska et al., 2019). Different quantity of the TPC and TFC and their antioxidant capacity in terms of TAC, TRP, and DPPH free radical scavenging capacity maybe because of the phytochemical polarity index and their association with solvent polarity index. Similar polarity index containing solvents can dissolve phytochemicals that have similar or close related polarity index (Raman et al., 2005). So, for highly active crude extract fractions specific solvent should be employed for the isolation and fractionation. The positive correlation between branches, flowers, leaves, and roots with diverse quantities of crude extracts indicate that different plant parts have a different amount of soluble phytochemicals that require a very specific solvent for isolation. Different biological compounds have different polarity and can be extracted with a solvent containing an appropriate polarity index (Musa et al., 2011; Jacotet-Navarro et al., 2018). Except for a few irregularities, the amount of TPC, TFC, TAC, and TRP was significantly increased with increasing polarity and abruptly decreasing at a very high polarity index such as water. It means that the plants have different biochemical compounds with a range of polarity. The amount and types of compounds with higher polarity might be very specific and scarce. The solvents/plant parts that violate the rule of increasing polarity with an increasing amount of biochemical maybe because of the higher or lower amount of these compounds with unique polarity. As we have performed only TPC and TFC, so further studies using HPLC and other important techniques are required to investigate the specific compounds using the highly efficient solvents and plant parts. Positive correlation among TPC, TAC, and TRP was observed as shown in the figure (Figs. 6A–6H), consistent with the previously reported work (Granato et al., 2018).

**Table 2:** The synergistic, antagonistic, or neutral effects of the solvents in (1:1) combination. B (branches); F (flowers); L (leaves) and R (roots). (+) indicate Synergistic, (−) indicate antagonistic and (0) indicate a neutral effect. The full form of the abbreviation used in the table: H:EA (n-hexane-ethyl-acetate); H:E (n-hexane-ethanol); M:C (methanol-chloroform); M:EA (methanol-ethyl-acetate); M:A (methanol-acetone); A:W (acetone-water) and M:W (methanol-water).

**Solvents used**	**Synergistic, antagonistic, or neutral effects of solvents**																							
	**% Extraction**				**TPC**				**TFC**				**TAC**				**TRP**				**DPPH**			
	**B**	**F**	**L**	**R**	**B**	**F**	**L**	**R**	**B**	**F**	**L**	**R**	**B**	**F**	**L**	**R**	**B**	**F**	**L**	**R**	**B**	**F**	**L**	**R**
**H:EA**	−	−	−	−	−	−	−	−	−	−	−	−	−	−	+	−	−	−	−	−	+	−	−	−
**H:E**	+	−	−	−	+	+	−	+	−	0	+	+	+	+	+	−	+	+	−	+	−	−	−	−
**M:C**	+	+	+	+	−	−	+	−	−	+	−	−	−	−	+	−	−	−	+	−	−	−	0	−
**M:A**	−	−	−	−	+	−	+	−	−	+	−	−	−	+	+	−	+	−	+	−	−	−	−	−
**M:A**	+	+	+	+	−	−	+	−	−	−	−	−	−	−	−	−	−	−	+	−	−	−	−	−
**A:W**	+	+	+	+	−	+	+	−	−	−	−	−	−	+	−	−	−	+	+	−	+	−	−	−
**M:W**	+	−	−	−	−	−	+	−	−	−	−	−	−	−	+	−	−	−	+	−	−	+	−	+

The (1:1, v/v) ratio combination of solvents showed synergistic, antagonistic, or neutral effects on the extraction efficiency, TPC, TFC, TRP, TAC, and DPPH free radical scavenging capacity (Table 2). Consistently, the synergistic, antagonistic, or neutral effects of these solvents on the antibacterial and antifungal activities have been previously reported (Ullah et al., 2017). This is because with 1:1 (v/v) ratio combinations, a unique and different polarity index is achieved that may or may not have successive polarity index containing compounds in the plant system.

## Conclusion

Different plant parts (extracted with a range of solvents with differnet polarity indexes) have a different amount of TPC, TFC, TRP, TAC, and DPPH free radical scavenging capacity. The other importance of the current study is that the selection of a specific solvent is very much important and the selection of an inappropriate solvent may cause false results. Furthermore, we can conclude that extracts of woad have a great amount of antioxidant, reducing power, and very low IC_50_ values based on the % DPPH free radical scavenging capacity . The identification of these biologically active crude extracts and fractions (based on the TPC, TFC, TRP, TAC, and DPPH free radical scavenging assays) are very important for the basic biological sciences, pharmaceutical applications, and future research for HPLC based active compounds isolation. Based on the current results, further investigations of these extracts as an antiprotozoal, anticancer, and cytotoxic agent are very important.

##  Supplemental Information

10.7717/peerj.7857/supp-1 Supplemental Information 1 Raw data

## Additional Information and Declarations

### Data Availability

The following information was supplied regarding data availability: The raw measurements are available in the Supplemental File.
